# Development of a decision flowchart to identify the patients need high-dose vancomycin in early phase of treatment

**DOI:** 10.1186/s40780-021-00231-w

**Published:** 2022-01-04

**Authors:** Ryo Yamaguchi, Hiroko Kani, Takehito Yamamoto, Takehiro Tanaka, Hiroshi Suzuki

**Affiliations:** 1 Department of Pharmacy, The University of Tokyo Hospital, Faculty of Medicine, The University of Tokyo, 7-3-1 Hongo, Bunkyo-ku, Tokyo, 113-8655 Japan; 2 The Education Center for Clinical Pharmacy, Graduate School of Pharmaceutical Sciences, The University of Tokyo, 7-3-1 Hongo, Bunkyo-ku, Tokyo, 113-0033 Japan

**Keywords:** Vancomycin, Decision tree analysis, Decision flowchart, High dose, Creatinine clearance, MRSA infection

## Abstract

**Background:**

The standard dose of vancomycin (VCM, 2 g/day) sometimes fails to achieve therapeutic concentration in patients with normal renal function. In this study, we aimed to identify factors to predict patients who require high-dose vancomycin (> 2 g/day) to achieve a therapeutic concentration and to develop a decision flowchart to select these patients prior to VCM administration.

**Methods:**

Patients who had an estimated creatinine clearance using the Cockcroft–Gault equation (eCCr) of ≥50 mL/min and received intravenous VCM were divided into 2 cohorts: an estimation set (*n* = 146, from April to September 2016) and a validation set (*n* = 126, from October 2016 to March 2017). In each set, patients requiring ≤2 g/day of VCM to maintain the therapeutic trough concentration (10–20 μg/mL) were defined as standard-dose patients, while those who needed > 2 g/day were defined as high-dose patients. Univariate and multivariate logistic regression analysis was performed to identify the predictive factors for high-dose patients and decision tree analysis was performed to develop decision flowchart to identify high-dose patients.

**Results:**

Among the covariates analyzed, age and eCCr were identified as independent predictors for high-dose patients. Further, the decision tree analysis revealed that eCCr (cut off value = 81.3 mL/min) is the top predictive factor and is followed by age (cut off value = 58 years). Based on these findings, a decision flowchart was constructed, in which patients with eCCr ≥81.3 mL/min and age < 58 years were designated as high-dose patients and other patients were designated as standard-dose patients. Subsequently, we applied this decision flowchart to the validation set and obtained good predictive performance (positive and negative predictive values are 77.6 and 84.4%, respectively).

**Conclusion:**

These results suggest that the decision flowchart constructed in this study provides an important contribution for avoiding underdosing of VCM in patients with eCCr of ≥50 mL/min.

**Supplementary Information:**

The online version contains supplementary material available at 10.1186/s40780-021-00231-w.

## Background

Vancomycin (VCM) is a glycopeptide antibiotic that is widely used for the treatment of infections caused by methicillin-resistant *Staphylococcus aureus* (MRSA) [1]. Because numerous number of reports have shown that the ratio of the area under the drug concentration–time curve over 24 h (AUC_24_, μg∙h/mL) to the minimum inhibitory concentration of pathogens (MIC, μg/mL), hereafter referred to as AUC_24_/MIC, is the best pharmacokinetic/pharmacodynamic (PK/PD) index to predict the clinical efficacy of VCM [2, 3], the latest Infectious Diseases Society of America (IDSA) guidelines [4] strongly recommends AUC-guided dosing to achieve an AUC_24_/MIC of 400–600 in place of conventional trough concentration (C_trough_)-guided dosing. However, it is sometimes time and cost consuming process to calculate AUC_24_ because it requires multiple blood sampling and pharmacokinetic analysis using dedicated software. Therefore, numbers of researchers have investigated the relationship between C_trough_ and AUC_24_ aiming to estimate AUC_24_ from single C_trough_. For instance, Clark et al. reported that C_trough_ of 12–18 μg/mL corresponded to AUC_24_ of 502–656 μg∙h/mL [5]. Further, several researchers have shown that C_trough_ of > 10 μg/mL was the significant predictive factor for AUC_24_ of > 400 μg∙h/mL in elderly patients [6, 7]. On the other hand, C_trough_ has also been extensively investigated as a predictor of nephrotoxicity of VCM, and Lodise et al. has reported that the risk of nephrotoxicity increases to 33% when the C_trough_ exceeds 20 μg/mL [8]. In addition, although several meta-analyses have investigated the superiority of AUC-guided dosing [9, 10], the most recent meta-analysis reported by Tsutsuura et al. [10] has not shown the superiority of AUC-guided dosing over C_trough_-guided dosing in both effectiveness and safety due to the large 95% confidential interval. Considering these reports, to achieve C_trough_ of 10–20 μg/mL would maintain certain clinical significance in the era of AUC-guided dosing.

Since more than 80% of intravenously administered VCM is excreted into the urine as unchanged form [11], the dosage of VCM should be individualized according to the renal function of the patient. Strategies for dosage adjustment of VCM in patients with impaired renal function, including patients on blood purification therapy, have been extensively investigated, and detailed dosing nomograms stratified by creatinine clearances (CCr) are available [12]. Whereas for patients with CCr of > 50 mL/min, 2 g/day (i.e., 1 g every 12 h), the standard dosage of VCM in package insert, is frequently selected as the initial dosage [13]. However, several studies recruiting critically ill patients or patients with heart failure have reported that augmented renal clearance (ARC), younger age, and sepsis status are the risk factors of subtherapeutic C_trough_ even after administration of the standard dosage (2 g/day) [14–17]. Although these risk factors may be applicable to non-critically patients or patients without heart failure from the pharmacokinetic point of view, but there have been insufficient reports to support this. Patients’ characteristics associated with subtherapeutic C_trough_ have also been explored using population PK (PPK) approach. Specifically, Yasuhara et al. have utilized population PK (PPK) approach and found that C_trough_ would be below 10 μg/mL in patients with normal renal function (CCr > 100–120 mL/min) even after administration of standard dose (1 g every 12 h) [18], though this estimation has not been in large population. Furthermore, Imai et al. applied a machine learning approach to determine optimal dosage for patients with estimated glomerular filtration rate (eGFR) of ≥50 mL/min/1.73m^2^ using eGFR, age, and BMI as predictive factors [19]. However, validation analysis indicated that C_trough_ of 33.5% of patients expected to be < 10 μg/mL. For other instance, Leu et al. proposed a dosing nomogram to achieve C_trough_ of 15–20 μg/mL and recommended 3 g/day of VCM for patients whose CCr is > 70 mL/min [20]. However, they also found that C_trough_ exceeded 20 μg/mL in 23.5% of patients whose VCM dosages were adjusted using this nomogram. Thus, it is necessary to develop methods to predict more accurately which patients would require a higher dose of VCM (> 2 g/day) to maintain the C_trough_ within the therapeutic range (10–20 μg/mL) in a patient population not limited to critically ill patients.

In this study, we aimed to identify the factors to predict patients with CCr of ≥50 mL/min who require > 2 g/day of VCM and to determine cut-off values. We developed a simple decision flowchart based on those cut off values to identify the patients who required high-dose (> 2 g/day) of VCM from the beginning of treatment and evaluated its usefulness using data from a validation cohort.

## Methods

### Study design and patients

This retrospective, observational study was performed at the University of Tokyo Hospital (Tokyo, Japan), a tertiary care, teaching hospital with 1217 beds.

Patients who received intravenous VCM from April 2016 to March 2017 were enrolled in the study. We included patients whose CCr estimated using the Cockcroft–Gault equation (eCCr) [21] was ≥50 mL/min immediately before VCM administration and whose steady state VCM C_trough_ was measured at least once. The exclusion criteria were defined as follows: (A) patients under 18 years of age, (B) first C_trough_ was measured within 2 days from the start of VCM administration [18, 22, 23], (C) VCM dosage was changed before the first C_trough_ measurement, and (D) renal function that fluctuated during VCM treatment. Fluctuation of renal function was defined as an increase in serum creatinine (SCr) by more than 1.5-fold from baseline within 7 days or more than 0.3 mg/dL from baseline within 48 h after the start of VCM administration, according to the Kidney Disease: Improving Global Outcomes (KDIGO) guidelines [24].

Patients who received intravenous VCM from April 2016 to September 2016 were assigned to the estimation set, which was used to develop a decision flowchart. Patients who received intravenous VCM from October 2016 to March 2017 were assigned to the validation set, which was used to validate the decision flowchart.

### Data collection

Age, sex, clinical department, body weight (BW), body mass index (BMI), SCr, initial VCM dosage, and VCM C_trough_ were extracted from patients’ medical records. The eCCr was calculated using the Cockcroft–Gault equation (Eq. 1) based on the SCr measured immediately before the intravenous administration of VCM: [21].

1 $$ \mathrm{eCCr}\ \left[\mathrm{mL}/\min \right]=\left(140-\mathrm{Age}\ \left[\mathrm{years}\right]\right)\times \mathrm{BW}\ \left[\mathrm{kg}\right]/\left(72\times \mathrm{SCr}\ \left[\mathrm{mg}/\mathrm{dL}\right]\right) $$

For female patients, the calculated value was multiplied by 0.85.

Because previous reports have shown that eCCr calculated using Eq. 1 in obese patients overestimates the actual CCr [25, 26], the adjusted ideal body weight (AIBW) [27] was calculated using the following equation (Eq. 2), and BW in Eq. 1 was substituted by AIBW when calculating eCCr in patient whose BMI was ≥30 kg/m^2^: [26].

2 $$ \mathrm{AIBW}\ \left[\mathrm{kg}\right]=\mathrm{IBW}\ \left[\mathrm{kg}\right]+0.4\times \left(\mathrm{BW}-\mathrm{IBW}\right) $$

where IBW represents the ideal BW calculated using the following equations (Eq. 3A, B):

3A $$ \mathrm{IBW}\ \left(\mathrm{Male}\right)\ \left[\mathrm{kg}\right]=50.0+0.9\times \left(\mathrm{height}\ \left[\mathrm{cm}\right]-152.4\right) $$

3B $$ \mathrm{IBW}\ \left(\mathrm{Female}\right)\ \left[\mathrm{kg}\right]=45.5+0.9\times \left(\mathrm{height}\ \left[\mathrm{cm}\right]-152.4\right) $$

### Definition of high-dose and standard-dose patients

In this study, patients were classified into two patient groups, high-dose patients and standard-dose patients, based on the VCM dosages needed to maintain C_trough_ above 10 μg/mL. Patients who needed no more than 2 g/day of VCM to maintain the C_trough_ of ≥10 μg/mL at steady-state were defined as standard-dose patients. Patients who needed more than 2 g/day of VCM (e.g., 1.5 g every 12 h or 1 g every 8 h) to maintain the C_trough_ of ≥10 μg/mL at steady-state were defined as high-dose patients. In this study, steady-state values of C_trough_ were considered to be those obtained after VCM administration at the same dosage for more than three days. In cases where the steady state C_trough_ was not measured and/or was in the subtherapeutic range (< 10 μg/mL), VCM dosages necessary to maintain C_trough_ within the therapeutic range (10–20 μg/mL) at steady-state were calculated using Bayesian estimation (BE). Calculations were conducted using the SHIONOGI-VCM-TDM E-edition ver. 2.04 (Shionogi Inc., Japan) software [28], and population PK parameters of VCM reported by Rodvold et al. were used [29].

### Decision-tree analysis

JMP 14.0 software (SAS Institute Inc., NC, USA) was used for the decision tree analysis based on recursive partitioning, to identify the factors predicting high-dose patients. The factors reached statistical significance in the univariate logistic regression analysis were included in the decision tree analysis. The partitioning was stopped when the number of patients in the node reaches < 20.

### Construction and validation of decision flowchart

Based on the final decision tree derived from the estimation set, a decision flowchart was constructed to identify the patients who needed high-dose VCM (> 2 g/day, e.g., 1.5 g every 12 h or 1 g every 8 h). Subsequently, the decision flowchart was applied to the validation set. The resulting sensitivity, specificity, positive predictive value (PPV), negative predictive value (NPV), positive likelihood ratio (PLR), and negative likelihood ratio (NLR) were calculated.

### Statistical analysis

To compare the characteristics of patients between the estimation set and validation set and between the high-dose and standard-dose patients, an unpaired *t*-test or Mann–Whitney *U*-test were used for the continuous variables, whereas a χ^2^-test were used for the categorical variables.

Univariate and multivariate logistic regression analyses were conducted to identify potential predictive factors for high-dose patients. The factors associated with subtherapeutic C_trough_ (≤10 μg/mL) in previous studies were included in the univariate analysis, and the factors reached statistical significance were employed as possible predictive factors in the decision tree analysis. Simultaneously, factors with *P* value < 0.1 in univariate analysis were subjected to a stepwise multivariate logistic regression analysis and the results were compared with those obtained in decision tree analysis. To ensure the independence of the explanatory variables found in the univariate analysis, the risk of multicollinearity was checked by examining the Pearson’s correlation coefficient between each pair of explanatory variables.

All tests for significance were two-tailed, and a *P* value of < 0.05 was considered statistically significant. The statistical analyses in this study were performed using SPSS version 24.0 software (IBM, Armonk, NY) except for the decision tree analysis.

## Results

### Characteristics of the patients

**Fig. 1:**
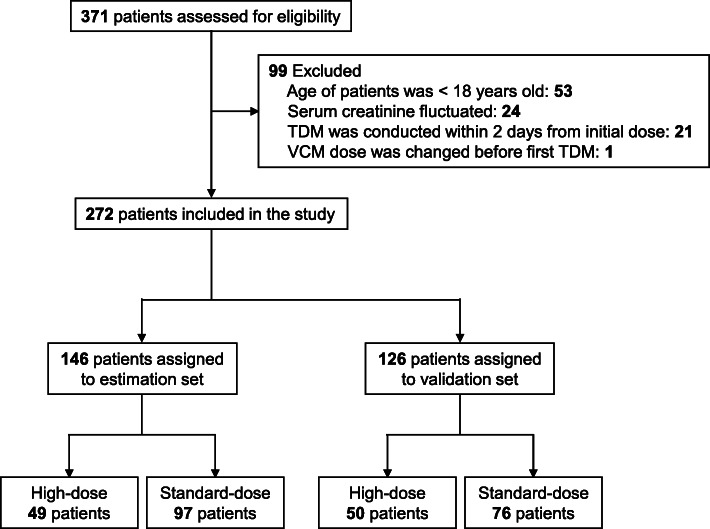
Selection flow of patients in this study

Of the 371 patients who received intravenous VCM during the study period and met the inclusion criteria, 272 patients were eligible for enrollment in the study. Of the 272 patients, 146 patients were assigned to the estimation set (high-dose patients, *n* = 49; standard-dose patients, *n* = 97), and 126 patients were assigned to the validation set (high-dose patients, *n* = 50; standard-dose patients, *n* = 76) (Fig. 1). Table 1 shows the characteristics of the patients assigned to the estimation and validation sets. As shown in Table 1, the characteristics of the patients were similar between the estimation and validation set although SCr and eCCr in validation set were significantly higher than in the estimation set. There were no significant differences in age, BW, BMI, days until first TDM, first C_trough_, clinical department, and suspected infection sites. BE was applied for 59 patients (40.4%) in the estimation set and 62 patients (49.2%) in the validation set, respectively.

**Table 1:** Baseline characteristics of patients in estimation and validation set

Characteristics		Estimation set (*n* = 146)	Validation set (*n* = 126)	*P* value
Male, n (%)		96 (65.8)	74 (58.7)	0.233^f^
Age [years]^a^		60.7 ± 15.0	57.8 ± 17.8	0.146^g^
Body weight [kg]^a^		58.3 ± 12.9	56.6 ± 14.1	0.615^g^
BMI [kg/m^2^]^a^		21.9 ± 3.9	21.5 ± 4.8	0.459^g^
sCr [mg/dL]^a^		0.69 ± 0.23	0.63 ± 0.24	0.025^g^
eCCr [mL/min]^a^		95.9 ± 43.8	109.6 ± 60.3	0.034^g^
Initial VCM dose				0.049^f^
> 2 g/day	n^b^ range [g/day]	11 (10/1/0) 2.5–4	19 (17/2/0) 2.25–3.75	
= 2 g/day	n^b^ range [g/day]	108 (0/107/1) NA^e^	77 (0/77/0) NA^e^	
< 2 g/day	n^b^ range [g/day]	27 (1/19/7) 1–1.6	30 (2/23/5) 0.5–1.5	
Days until First TDM [days]^c^		4 (2–7)	3 (2–6)	0.334^h^
First C_trough_ [μg/mL]^a^				
All patients		13.0 ± 6.0	12.1 ± 5.4	0.185^g^
> 2 g/day		13.2 ± 3.5	13.6 ± 4.1	0.821^g^
= 2 g/day		13.3 ± 6.1	11.6 ± 5.5	0.056^g^
< 2 g/day		11.8 ± 6.6	12.3 ± 5.7	0.743^g^
BE conducted, n (%)		59 (40.4)	62 (49.2)	0.146^f^
Clinical department, n				0.890^f^
Hematology		27	19	
Gastroenterology		13	10	
Cardiology		12	12	
Orthopedics		11	9	
Neurosurgery		12	15	
Cardiac surgery		9	5	
Other		62	56	
Suspected infection sites, n				0.246^f^
CR-BSI^d^		25	27	
Febrile neutropenia		25	16	
Surgical site infection		24	20	
Pneumonia		12	10	
Peritonitis		10	1	
Cholangitis		9	6	
Urinary tract infection		6	4	
Cellulitis		5	5	
Others		30	37	

^a^Data are shown as mean ± standard deviation (SD)

^b^Numbers in parentheses indicate the number of patients with dosing intervals of 8 h, 12 h, and others from the left, respectively

^c^Data are shown as median (range)

^d^Catheter-related blood stream infection

^e^Not applicable because all patients in =2 g/day group uniformly received 2 g/day of VCM

^f^χ^2^-test

^g^Unpaired student’s *t*-test

^h^Mann–Whitney *U*-test

### Univariate and multivariate logistic regression analysis

Table 2 summarizes the characteristics of patients assigned to the estimation set. Significant differences in age, BW, BMI, SCr, and eCCr were observed between the high-dose and standard-dose groups. Because a strong positive correlation (*r* = 0.842) between BW and BMI were observed using the Pearson’s correlation test conducted prior to multivariate logistic regression analysis, we entered these two variables into a multivariate logistic regression analysis to check the risk of multicollinearity. The results were similar regardless of whether BW or BMI were entered into the analysis, and age and eCCr were independently associated with high-dose patients.

**Table 2:** Univariate and multivariate logistic regression analysis in estimation set

Characteristics	All patients (*n* = 146)	High-dose (*n* = 49)	Standard-dose (*n* = 97)	*P* value	
				Univariate	Multivariate
Male, n (%)	96 (65.8)	31 (63.3)	65 (67.0)	0.653	
Age [years]^a^	60.7 ± 15.0	52.0 ± 15.2	65.0 ± 12.9	< 0.001	0.020
Body weight [kg]^a^	58.3 ± 12.9	63.1 ± 13.7	55.8 ± 11.9	0.002	–
BMI [kg/m^2^]^a^	21.9 ± 3.9	23.1 ± 4.3	21.3 ± 3.5	0.011	–
SCr [mg/dL]^a^	0.69 ± 0.23	0.62 ± 0.19	0.73 ± 0.24	0.003	–
eCCr [mL/min]^a^	95.9 ± 43.8	123.0 ± 40.4	82.2 ± 38.9	< 0.001	0.001
Initial VCM dose, n^b^				< 0.001^d^	
> 2 g/day	11 (10/1/0)	11 (10/1/0)	0 (0/0/0)		
=2 g/day	108 (0/107/1)	34 (0/34/0)	74 (0/73/1)		
< 2 g/day	27 (1/19/7)	4 (1/2/1)	23 (0/17/6)		
Day until first TDM [days]^c^	4 (2–7)	3 (2–7)	4 (3–6)	0.213^e^	
First C_trough_ [μg/mL]^a^					
All patients	13.0 ± 6.0	8.2 ± 3.8	15.5 ± 5.5	< 0.001^f^	
> 2 g/day	13.3 ± 3.5	13.3 ± 3.5	–	–	
=2 g/day	13.3 ± 6.1	6.8 ± 2.1	16.3 ± 4.9	< 0.001^f^	
< 2 g/day	11.8 ± 6.7	5.8 ± 3.5	12.8 ± 6.5	0.047^f^	
BE conducted, n (%)	59 (40.4)	33 (67.3)	26 (26.8)	< 0.001^d^	

^a^Data are shown as mean ± standard deviation (SD)

^b^Numbers in parentheses indicate the number of patients with dosing intervals of 8 h, 12 h, and others from the left, respectively

^c^Data are shown as median (range)

^d^χ^2^-test

^e^Mann–Whitney *U*-test

^f^Unpaired student’s *t*-test

*BMI* body mass index, *SCr* serum creatinine, *eCCr* estimated creatinine clearance, *C*_*trough*_ trough concentration of VCM, *BE* Bayesian estimation

### Decision tree analysis

**Fig. 2:**
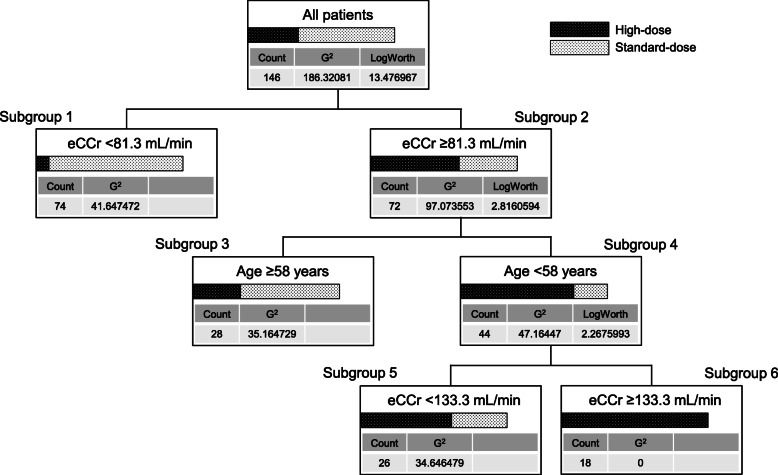
Final decision tree. Final decision tree with three layer and two predictive factors (age and eCCr) is shown. The cut off value for the split is determined by maximizing the LogWorth. G^2^ value indicates randomness in each subgroup (G^2^ = 0 means perfect fit). The decision tree analysis revealed that eCCr is the top predictive factor and followed by age

The final decision tree with three layer is shown in Fig. 2. Among the four factors assessed (age, BW, sCr, and eCCr), age and eCCr were identified as significant predictive factors and these results are consistent with those of multivariate logistic regression analysis (Table 2). The patients were finally classified into four subgroups (subgroup 1, 3, 5, and 6, Fig. 2) using age and eCCr. JMP software automatically classified patients in the subgroups 1, 3 and 5, 6 as standard-dose and high-dose patients, respectively. The sensitivity, specificity, PPV, NPV, PLR, and NLR for estimation set were 69.4, 89.7, 77.3, 85.3%, 6.74, and 0.34, respectively (see Additional file 1).

### Construction and validation of decision flowchart

**Fig. 3:**
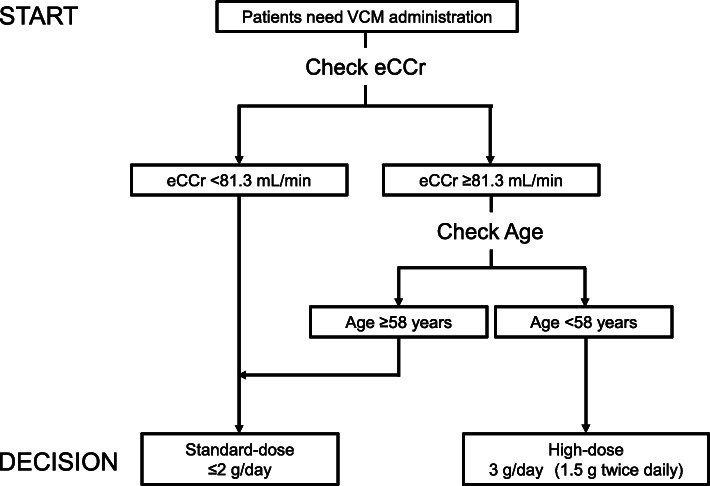
Decision flowchart for selecting patients who need high-dose VCM. This decision flowchart is constructed to be used for patients with eCCr of ≥50 mL/min. Patients with age of < 58 years and eCCr of 81.3–133.3 mL/min are at higher risk of overdosing than patients with age of < 58 years and eCCr of > 133.3 mL/min when received 3 g/day of VCM. Thus, for these patients, 2 g/day of dose may be considered depending on the patient’s condition

Based on the results of decision tree analysis, we constructed a practical decision flowchart based on eCCr, and age (Fig. 3). In the final decision tree, patients in subgroup 4 (patients with eCCr of ≥81.3 mL/min and age of < 58 years) were further split into subgroups 5 and 6 using eCCr of 133.3 mL/min as cut off value (Fig. 2). However, the decision flowchart did not further split the subgroup 4 because JMP software automatically classified subgroups 5 and 6 as high-dose group.

We then applied the decision flowchart to the validation set. The summary of patients’ characteristics in the validation set are presented in Tables 1 and 3. Statistically significant differences were observed in age, BW, SCr, CCr, and initial VCM dose between high-dose and standard-dose patients within the validation set (Table 3), and these observations were similar to those observed within the estimation set. The sensitivity, specificity, PPV, NPV, PLR, and NLR of this decision flowchart for validation set were 76.0, 85.5, 77.6, 84.4%, 5.24, and 0.28, respectively (see Additional file 1).

**Table 3:** Characteristics of patients classified in validation set

Characteristics	All patients (*n* = 126)	High-dose (*n* = 50)	Standard-dose (*n* = 76)	*P* value
Male, n (%)	74 (58.7)	25 (50.0)	49 (64.5)	0.331^d^
Age [years]^a^	57.8 ± 17.8	46.5 ± 14.6	65.1 ± 15.5	< 0.001^e^
Body weight [kg]^a^	56.6 ± 14.1	60.8 ± 15.1	53.9 ± 12.7	0.009^e^
BMI [kg/m^2^]^a^	21.5 ± 4.8	22.5 ± 5.7	20.9 ± 4.0	0.096^e^
SCr [mg/dL]^a^	0.63 ± 0.24	0.54 ± 0.20	0.69 ± 0.25	< 0.001^e^
eCCr [mL/min]^a^	109.6 ± 60.3	145.8 ± 56.4	85.9 ± 50.3	< 0.001^e^
Initial VCM dose, n^b^				< 0.001^d^
> 2 g/day	19 (17/2/0)	16 (14/2/0)	3 (3/0/0)	
=2 g/day	77 (0/77/0)	29 (0/29/0)	48 (0/48/0)	
< 2 g/day	30 (2/23/5)	5 (1/4/0)	25 (1/19/5)	
Days until first TDM [days]^c^	3 (2–6)	3 (3–5)	3 (2–6)	0.962^f^
First C_trough_ [μg/mL]^a^				
All patients	12.1 ± 5.4	8.5 ± 4.2	14.4 ± 4.8	< 0.001^e^
> 2 g/day	13.6 ± 4.1	13.0 ± 4.0	16.8 ± 4.0	0.140^e^
=2 g/day	11.6 ± 5.5	6.8 ± 2.1	14.6 ± 4.8	< 0.001^e^
< 2 g/day	12.3 ± 5.7	4.6 ± 2.1	13.9 ± 4.9	< 0.001^e^
BE conducted, n (%)	62 (49.2)	30 (60.0)	32 (42.1)	0.049^d^

^a^Data are shown as mean ± standard deviation (SD)

^b^Numbers in parentheses indicate the number of patients with dosing intervals of 8 h, 12 h, and others from the left, respectively

^c^Data are shown as median (range)

^d^χ^2^-test

^e^Unpaired student’s *t*-test

^f^Mann–Whitney *U*-test

*BMI* body mass index, *SCr* serum creatinine, *eCCr* estimated creatinine clearance, *C*_*trough*_ trough concentration of VCM, *BE* Bayesian estimation

## Discussion

In this study, we developed a simple decision flowchart based on age and eCCr to predict patients who need high-dose (3 g/day) VCM. When applied to the validation set, this decision flowchart demonstrated successful prediction of patients requiring high-dose VCM to maintain the steady-state C_trough_ of ≥10 μg/mL.

In our study population, 38.5% (106/275) of patients were classified as high-dose patients. This observation indicates that a significant proportion of patients with eCCr of greater than 50 mL/min are at risk of underdosing (i.e., C_trough_ < 10 μg/mL) when treated with a standard dose of VCM (2 g/day). In a previous study, Maki et al. reported that 31% of patients with eCCr ≥50 mL/min failed to achieve C_trough_ of ≥10 μg/mL after intravenous administration of the standard dose of VCM (2 g/day) [13]. Rosini et al. also reported that approximately 40% of patients failed to achieve C_trough_ of ≥10 μg/mL 36 h after an initial intravenously administered VCM dose of 15 mg/kg every 12 h, which approximates to 2.6 g/day based on an average BW (87 kg) [30]. Similar results were observed in a study conducted in patients with eGFR of ≥90 mL/min/1.73 m^2^ [31]. These results are all consistent with those of the present study and indicate that a large number of patients need high-dose VCM to achieve the C_trough_ of ≥10 μg/mL.

There have been several reports indicating a possible relationship between younger age and lower C_trough_. Revilla et al. reported that only 33.4% of critically ill patients under 65 years of age and with eCCr of > 60 mL/min could attain the target PK/PD index (AUC_24_/MIC > 400) after intravenous administration of VCM at the dose of 2 g/day [32]. In addition, Ishii et al. reported that younger age (< 50 years) was associated with subtherapeutic C_trough_ after dosage adjustment based on individual eGFR [33]. Interestingly, in our study population, age is a significant predictor only in patients with eCCr ≥81.3 mL/min (Fig. 2). Although the underlying mechanism of the eCCr-dependent effect of age observed in our study population is unclear, we believe it may be partially attributable to overestimation of renal function in elderly patients with high eCCr. Previous studies have shown that creatinine production tends to decrease owing to loss of muscle mass in elderly patients and consequently, eCCr calculated from SCr tends to overestimate the actual renal function [34]. In elderly patients with eCCr ≥81.3 mL/min, low SCr could be reflecting loss of muscle mass rather than increased renal excretion; thus, the discrepancy between eCCr and actual renal function in these patients would be larger than that in patients with eCCr < 81.3 mL/min.

Although there were some differences in patient characteristics between the estimation set and validation set (Table 1), PPV and NPV were 77.6 and 84.4% respectively, and these values were comparable to those in the estimation set (69.4 and 89.7% respectively). This observation seems to support the preferable predictive performance and the robustness of the decision flowchart developed in this study. When the predictive performance in this study is compared with those in previous report by Imai et al. [19], our decision flowchart showed a lower risk of underdosing (10.3% vs 33.5%) and a higher risk of overdosing (30.6% vs 15.8%). This indicates that our decision flowchart tends to overestimate the dosage compared to the algorithm reported by Imai et al. Although the reason why our decision flowchart tends to overestimate the dosage is unclear, one possible explanation is that patients in subgroup 5 (patients with age of < 58 years and eCCr of 81.3–133.3 mL/min) were uniformly classified as high-dose patients. When patients classified as subgroup 5 (age < 58 years, CCr 81–133) were judged as standard-dose, the PPV and NPV changed to 86.2 and 74.2%, respectively (see Additional file 1). This suggests that about 15% of patients are at the risk of overdosing, while about 25% are at the risk of underdosing. These values are similar to those reported by Imai et al., although the risk of underdosing is somewhat lower in our decision flowchart.

Since this study focused on the pharmacokinetic evaluation, we excluded patients with fluctuating renal function from this study. Although there were no patients who were classified as high-dose group based on our decision flowchart and actually received > 2 g/day of VCM among the excluded patients due to fluctuating renal function (data not shown), the risk of VCM-induced kidney injury remains unclear when our decision flowchart is applied to daily clinical practice. Therefore, careful consideration should be taken to avoid overdosing when applying our decision flowchart to patients receiving VCM, especially those classified in subgroup 5, the subgroup with poor predictivity. As shown in Fig. 2, the proportions of high-dose and standard-dose patients in subgroup 5 are 61.5 and 38.5%, respectively. Therefore, if 3 g/day of VCM is uniformly selected for patients classified into subgroup 5, approximately 40% of patients are at the risk of overdosing. This indicate that patients in subgroup 5 (patients with age of < 58 years and eCCr of 81.3–133.3 mL/min) are at higher risk of overdosing compared to patients in subgroup 6 (patients with age of < 58 years and eCCr of > 133.3 mL/min). Thus, for patients in subgroup 5, 2 g/day of dose may be considered depending on the patient’s condition (e.g., dehydration, concomitant use of calcineurin inhibitors, aminoglycosides, or piperacillin/tazobactam). In addition, because it has been reported that VCM-induced kidney injury tends to occur after the fourth day from initial administration [35, 36], the risk of VCM-induced kidney injury would be minimized by performing TDM on the third or fourth day of treatment and adjusting the dosage.

In this study, we defined therapeutic C_trough_ as 10–20 μg/mL based on the previous pharmacokinetic studies [5–7]. However, in the latest IDSA guidelines [4], an aggressive C_trough_ (> 15 μg/mL) is no longer recommended for serious MRSA infections to minimize the risk of nephrotoxicity. In addition, Oda et al. recently reported that the estimated C_trough_ needed to maintain the AUC_24_ at 400–560 in patients with eGFR of ≥60 mL/min/1.73 m^2^ is 9.3–14.8 μg/mL [37]. Taking these recent literatures into consideration, C_trough_ of 10–15 μg/mL has a demonstrated clinical value as a predictive index of AUC_24_/MIC, although C_trough_ of 15–20 μg/mL may increase the risk of acute kidney injury and should be avoided. Therefore, careful attention should be paid when interpreting the results of this study in clinical settings. In our study population, the mean C_trough_ in high-dose patients at the dose of 2 g/day was 6.8 μg/mL in both estimation and validation sets. Therefore, we estimate that if the dosage is increased to 3 g/day, the C_trough_ would still be controlled within 10–15 μg/mL. For these reasons, 3 g/day of VCM would be recommended for patients classified as high-dose patient by our decision flowchart (Fig. 3). In routine practice, 1 g of VCM every 8 h (thrice a day) or 1.5 g every 12 h (twice a day) is the usual dosage regimen to administer 3 g/day total VCM, considering the ease of administration and the dosage unit of VCM (0.5 g/vial). However, based on the principle of pharmacokinetics, twice a day administration (1.5 g every 12 h) achieves lower C_trough_ than thrice a day administration (1 g every 8 h); thus, it seems safer to choose 1.5 g every 12 h.

There are several limitations to our study. First, this study was a single-center, retrospective, observational study. Therefore, the possible interference of unintentional selection biases may exist; hence, the generalizability of our results should be confirmed in future studies. Second, clinical efficacy and safety were not evaluated in this study. Nevertheless, since the mean C_trough_ in high-dose patients (VCM > 2 g/day) was below 15 μg/mL (13.3 ± 3.5 and 13.1 ± 3.9 μg/mL for the estimation and validation sets, respectively (Tables 2, 3), the risk of nephrotoxicity would be acceptable in clinical settings. Third, sepsis status was not evaluated as a possible predictive factor of subtherapeutic C_trough_ in the decision-tree analysis due to the difficulty in diagnosing sepsis from chart review. Therefore, predictive value of sepsis status should be evaluated in future studies. Fourth, BE is applied for half of the patients to discriminate high-dose patients from standard-dose patients. However, considering that only patients with eCCr ≥50 mL/min were included, and the first C_trough_ was measured at least 3 days after the start of treatment, we believe that the results of BE are reliable. Fifth, no patients included in this study received loading dose. Although we expect that the decision flowchart is applicable to patients who received loading dose since steady-state C_trough_ was utilized in this study, further studies are needed to elucidate whether our results is applicable to patients who received loading dose.

## Conclusion

We developed, and validated a decision flowchart using eCCr and age to predict which patients would need high-dose VCM (3 g/day, e.g., 1.5 g every 12 h). This decision flowchart will provide an important contribution for avoiding underdosing of VCM in patients with eCCr of ≥50 mL/min.

## Supplementary Information

**Additional file 1.**

## Data Availability

The datasets used and/or analyzed during the current study are available from the corresponding author on reasonable request.

## Abbreviations

AIBW: Adjusted ideal body weight
ARC: Augmented renal clearance
AUC-ROC: Areas under the receiver operating characteristic curve
AUC_24_: Area under the drug concentration–time curve over 24 h
AUC_24_/MIC: Ratio of area under the drug concentration–time curve over 24 h to minimum inhibitory concentration of pathogens
BE: Bayesian estimation
BMI: Body mass index
BW: Body weight
CCr: Creatinine clearances
CI: Confidential interval
C_trough_: Trough concentration of vancomycin
eCCr: Estimated creatinine clearance using the Cockcroft–Gault equation
eGFR: Estimated glomerular filtration rate
IBW: Ideal body weight
KDIGO: Kidney Disease Improving Global Outcomes
MIC: Minimum inhibitory concentration of pathogens
MRSA: Methicillin-resistant *Staphylococcus aureus*
NLR: Negative likelihood ratio
NPV: Negative predictive value
PK/PD: Pharmacokinetic/pharmacodynamic
PLR: Positive likelihood ratio
PPV: Positive predictive value
ROC: Receiver operating characteristic
SCr: Serum creatinine
VCM: Vancomycin
