# Supplementary material for: Development of a decision flowchart to identify the patients need high-dose vancomycin in early phase of treatment
Source: J Pharm Health Care Sci. 2022 Jan 4;8:3. doi: 10.1186/s40780-021-00231-w (PMC8725522; doi:10.1186/s40780-021-00231-w)
Supplement: Supplementary file 1 — Additional file 1. [file 40780_2021_231_MOESM1_ESM.docx]

Supplemental table 1. Contingency matrix for estimation set (per final decision tree)

|  | | Decision flowchart | |  |
| --- | --- | --- | --- | --- |
|  |  | High-dose | Standard-dose | Total |
| Observed | High-dose | 34 | 15 | 49 |
|  | Standard-dose | 10 | 87 | 97 |
| Total | | 44 | 102 | 146 |

Prediction performance are as following: sensitivity, 69.4%; specificity, 89.7%; positive predictive value (PPV), 77.3%; negative predictive value (NPV), 85.3%; positive likelihood ratio (PLR), 6.74; negative likelihood ratio (NLR), 0.34.

Supplemental table 2. Contingency matrix for validation set (per decision flowchart)

|  | | Decision flowchart | |  |
| --- | --- | --- | --- | --- |
|  |  | High-dose | Standard-dose | Total |
| Observed | High-dose | 38 | 12 | 50 |
|  | Standard-dose | 11 | 65 | 76 |
| Total | | 49 | 77 | 126 |

Prediction performance are as following: sensitivity, 76.0%; specificity, 85.5%; positive predictive value (PPV), 77.6%; negative predictive value (NPV), 84.4%; positive likelihood ratio (PLR), 5.24; negative likelihood ratio (NLR), 0.28.

Supplemental table 3. Contingency matrix for validation set (subgroup 5 is judged as standard-dose group)

|  | | Decision flowchart | |  |
| --- | --- | --- | --- | --- |
|  |  | High-dose | Standard-dose | Total |
| Observed | High-dose | 25 | 25 | 50 |
|  | Standard-dose | 4 | 72 | 76 |
| Total | | 29 | 97 | 126 |

Prediction performance are as following: sensitivity, 50.0%; specificity, 94.7%; positive predictive value (PPV), 86.2%; negative predictive value (NPV), 74.2%; positive likelihood ratio (PLR), 9.43; negative likelihood ratio (NLR), 0.53.
